# Adrenal Gland Ultrasonographic Measurements and Plasma Hormone Concentrations in Clinically Healthy Newborn Thoroughbred and Standardbred Foals

**DOI:** 10.3390/ani11061832

**Published:** 2021-06-19

**Authors:** Eleonora Lauteri, Jole Mariella, Francesca Beccati, Ellen Roelfsema, Carolina Castagnetti, Marco Pepe, Tanja Peric, Olimpia Barbato, Marta Montillo, Stefanie Rouge, Francesca Freccero

**Affiliations:** 1 Department of Veterinary Medicine, University of Perugia, Via San Costanzo 4, 06126 Perugia, Italy; francesca.beccati@unipg.it (F.B.); marco.pepe@unipg.it (M.P.); olimpia.barbato@unipg.it (O.B.); stefanie.rouge@hotmail.it (S.R.); 2 Clinèquine, VetAgro Sup, University of Lyon, 1 Avenue Bourgelat, Marcy-l’Etoile, 69280 Lyon, France; 3 Department of Veterinary Medical Sciences, University of Bologna, Via Tolara di Sopra 50, Ozzano dell’Emilia, 40064 Bologna, Italy; jole.mariella2@unibo.it (J.M.); carolina.castagnetti@unibo.it (C.C.); francesca.freccero2@unibo.it (F.F.); 4 Department of Equine Sciences, Faculty of Veterinary Medicine, Utrecht University, Yalelaan 1, 3584 CL Utrecht, The Netherlands; E.Roelfsema@uu.nl; 5 Health Science and Technologies Interdepartmental Center for Industrial Research (HST-ICIR), University of Bologna, 40126 Bologna, Italy; 6 Department of Agricultural, Food, Environmental and Animal Sciences, University of Udine, Via Sondrio 2/B, 33100 Udine, Italy; tanja.peric@uniud.it (T.P.); marta.montillo@uniud.it (M.M.)

**Keywords:** adrenal glands, ultrasound, neonatal foals, adrenal hormones, ACTH, age, sex, breed, weight

## Abstract

**Simple Summary:**

The hypothalamic–pituitary–adrenal axis regulates many physiologic and metabolic functions and plays a central role in the inflammatory response to illness. Clinically, hypothalamic–pituitary–adrenal axis function can be evaluated by combined assessment of adrenal hormones, adrenocorticotropin plasma concentrations and ultrasonographic examination of the adrenal glands in other species. Multiple individual variables have been demonstrated to affect ultrasonographic measurements of adrenal glands in several species. Ultrasonographic measurements of the adrenal glands and plasma concentration of adrenocorticotropin, cortisol, dehydroepiandrosterone, progesterone, aldosterone and adrenocorticotropin/adrenal hormone ratios were assessed and compared between Thoroughbred and Standardbred healthy neonatal foals. The effect of age, weight and sex on ultrasonographic measurements of the adrenal glands was also investigated. The width of whole adrenal glands and the medulla differed significantly between Thoroughbreds and Standardbreds. Cortisol and adrenocorticotropin were, respectively, higher and lower in Thoroughbreds than in Standardbreds. The cortisol/dehydroepiandrosterone ratio was higher in Thoroughbreds than in Standardbreds. The results of this study provide reference ranges for healthy neonatal Thoroughbred and Standardbred foals and suggest the presence of breed-related differences in ultrasonographic measurements, plasmatic cortisol and adrenocorticotropin concentrations. The higher cortisol/dehydroepiandrosterone ratio of Thoroughbred foals may suggest a different response to stress and environmental stimulation between the two breeds.

**Abstract:**

Adrenal hormones, ACTH plasma concentrations and the ultrasonographic evaluation of the adrenal glands are considered complementary in clinical evaluations of the hypothalamic–pituitary–adrenal (HPA) axis function in several species. In dogs, age, size and weight have a significant effect on the ultrasonographic size of the adrenal glands. In neonatal foals, ultrasonographic evaluation and measurements of the adrenal glands have been demonstrated to be reliable; however, the effect of individual variables on ultrasonographic features has not been investigated, and the clinical usefulness of adrenal gland ultrasonography is still not known. The aims of this study were: (i) to provide and compare adrenal glands ultrasound measurements in healthy newborn Thoroughbred and Standardbred foals, and assess any effect of age, weight and sex on them; (ii) to assess and compare ACTH and steroid hormone concentrations in healthy neonatal foals of the two breeds. Venous blood samples and ultrasonographic images of the adrenal glands were collected from 10 healthy neonatal Thoroughbred and 10 healthy neonatal Standardbred foals. Ultrasonographic measurements of the adrenal glands were obtained and adrenocorticotropin (ACTH), cortisol, dehydroepiandrosterone (DHEA), progesterone (P4) and aldosterone plasma concentrations were assessed. The ACTH/cortisol, ACTH/progesterone, ACTH/aldosterone, ACTH/DHEA and cortisol/DHEA ratios were calculated. A significant positive correlation was found between the height of the right adrenal gland and the foal’s weight; the width of the right and left adrenal gland and the medulla was significantly lower in Thoroughbreds than in Standardbreds. Cortisol and ACTH plasma concentrations were significantly higher and lower, respectively, in Thoroughbreds compared with Standardbreds. The cortisol/DHEA ratio was significantly higher in Thoroughbreds. This study provides reference ranges for neonatal Thoroughbred and Standardbred foals, and suggests the presence of breed-related differences in ultrasonographic adrenal gland measurements, plasmatic cortisol and ACTH concentrations. The higher cortisol/DHEA ratio detected in Thoroughbred foals could suggest a different response to environmental stimulation in the two breeds.

## 1. Introduction

The adrenal glands are part of the hypothalamus–pituitary–adrenal (HPA) axis and its effective organ [1]. This axis, together with the renin–angiotensin–aldosterone system (RAAS), is involved in the homeostasis of the organism, regulating many physiologic and metabolic functions along with the inflammatory responses to illness [2,3,4]. It is fundamental for the process of adaptation to the extrauterine environment and, in horses, cortisol, together with progesterone (4-pregnen-3,20-dione or P4), and dehydroepiandrosterone (DHEA) are extremely important in the transition from intrauterine to extrauterine life [5,6,7,8]. Normally, plasma cortisol concentrations increase in the last 3 days of gestation in horses; contrarily, P4 decreases significantly in the first 24 h after delivery, reaching concentrations so low that they are difficult to determine [9]. DHEA, as a fetal component of the feto-placental unit for estrogen production, is secreted in large quantities by the gonads and adrenals during pregnancy; its concentration is highly variable in the first day of life and it significantly decreases in the days thereafter [10,11,12,13].

Thus, plasma cortisol and P4 concentrations at birth are used as indicators of foal maturity and health [9,14].

In neonatal foals, several studies have demonstrated that HPA axis dysfunction frequently develops during critical illness in neonatal foals, contributing substantially to morbidity and mortality associated with the primary disease [15,16].

Evaluation of the HPA axis function is usually achieved through the adrenocorticotropin (ACTH) stimulation test; however, albeit less accurate, the ACTH/cortisol ratio is widely used and provides valuable information [12,15,17,18,19]. Recently, it has been suggested that in addition to cortisol, aldosterone and multiple steroids are also involved in the response of the HPA axis to illness and that their plasma concentrations vary significantly in the case of HPA axis dysfunction, providing useful clinical information [20]. DHEA is strongly suggested to be linked to health maintenance in humans and in adult miniature Yucatan swine [21]; recently, cortisol, DHEA and their ratio have been defined to be related to allostatic load and resilience in adult pigs and cows [22,23,24,25].

In small-animal and human medicine, the assessment of adrenal hormones and ACTH plasma concentrations and the ultrasonographic evaluation of the adrenal glands are considered complementary in the evaluation of HPA axis function [26,27,28,29]. In these species, ultrasonographic evaluation of adrenal glands is considered a sensitive and specific screening method for assessing the adrenal glands’ size and appearance [30,31,32,33]. In companion species, it is used in the diagnosis of several disease such as hyperthyroidism, hypo-and hyperadrenocorticism, hyperaldosteronism and endocrine tumors [28,34]. Interestingly, in dogs, age, breed, size and weight are reported to have a significant effect on the ultrasonographic size of the adrenal glands [35,36], and these variables are taken into account during ultrasonographic evaluations of the adrenal glands [37,38].

In adult horses, transrectal ultrasonography allows an adequate visualization of the left adrenal gland [39], and in neonatal foals, ultrasonographic examination of the adrenal glands has been demonstrated to be feasible [40]. Recently, ultrasonographic measurements of the height of the left and right whole adrenal glands, the width of the left and right whole adrenal glands and the width of the left and right adrenal medulla were obtained using a convex probe in a transabdominal transverse–oblique plane in neonatal foals and were demonstrated to be reliable [41]. However, the clinical value of ultrasonography of the adrenal glands and the effect of age, weight, breed and sex on the ultrasonographic features of the adrenal glands are still not known in neonatal foals.

The aims of this cohort study were: (i) to provide and compare ultrasound measurements of the adrenal glands in healthy neonatal Thoroughbred and Standardbred foals and assess any effect of age, weight and sex; (ii) to assess and compare ACTH and steroid plasma hormone concentrations in healthy neonatal foals of the two breeds.

## 2. Materials and Methods

### 2.1. Foals

Healthy neonatal Thoroughbred (TB) and Standardbred (SB) foals aged less than 72 h were included in the study.

The TB foals were born on a farm close to the Veterinary Teaching Hospital of the University of Perugia; the SB foals had been foaled by mares referred for attended delivery to the Equine Perinatology Unit of the University of Bologna during the 2016–2018 foaling season. All foals were privately owned. Owners provided informed consent to the procedures. Foals less than 72 h old undergoing routine clinical monitoring were enrolled on a convenience basis, randomly selected based on the willingness of the client to participate in the study.

The criteria for a foal’s inclusion were: normal pregnancy, gestational length >320 days [41], absence of treatment during pregnancy and eutocic delivery, normal physical examination, hematology (white blood cell count and leukogram, red blood cell and platelet counts, packed cell volume, and hemoglobin concentration) and biochemistry (total proteins, albumin, total and direct bilirubin, glucose, urea, creatinine, alkaline phosphatase, lactate dehydrogenase, creatine kinase, gamma glutamyl transferase, aspartate aminotransferase, electrolyte concentrations and fibrinogen) within the normal range, and serum IgG concentrations >800 mg/dL at 18–24 h of life. The mare’s age and parity, and the foal’s month of birth, sex, age and weight were recorded for the two groups.

In both settings, foals were housed with their dams and free to nurse. They were turned out during the day and stabled at night. The environment and the habits of the foals and the mare remained unvaried for the first week of life of the foal. All the foals underwent a daily physical examination during the observation period of about 7 days.

### 2.2. Study Protocol

Each foal underwent an ultrasound exam of the adrenal glands and determination of the adrenal hormone concentrations once within the first 72 h of life. Ultrasound was performed as adjunctive part of a routine ultrasonographic examination of umbilical remnants in the neonates. Before the ultrasound evaluation, a blood sample was harvested for routine blood work, and an aliquot was used for hormone concentration determination. The blood samples were collected at the same time for both groups (8.00 or 9.00 a.m.).

To reduce stress to the foals, a maximum of 3 people entered the stable each time, waiting about 10–15 min for conditioning/acclimatation of the foals.

### 2.3. Blood Samples

Blood samples were collected by jugular venipuncture into plastic tubes containing anticoagulant and a gel clot activator for routine CBC and biochemistry analysis. For the study purpose, an aliquot plasma sample for each foal was centrifuged at 4000× *g* for 5 min within 3 h of collection and stored at −80 °C until subsequent hormone determination.

### 2.4. Adrenal Gland Ultrasonography

Two different operators (E.L.; F.F.) with previous experience in ultrasonographic examination of the adrenal glands [41] performed the exams in the Thoroughbred and Standardbred foals; 1 operator worked on the Thoroughbreds and 1 on the Standardbreds.

**Figure 1:**
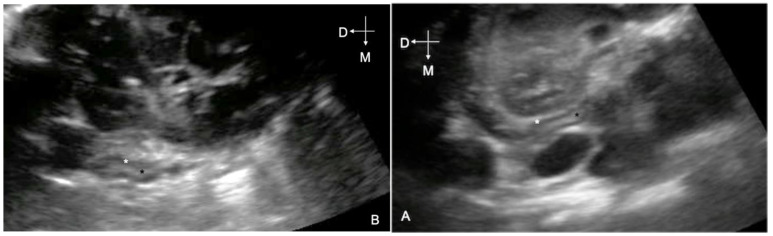
Ultrasound image of the right (**A**) and the left (**B**) adrenal gland of a healthy Thoroughbred foal. The figure illustrates the normal aspect of the adrenal glands; the cortex and medulla of each adrenal gland are indicated by white and black asterisks, respectively.

Before or after assessment of the umbilical remnants, ultrasonographic videos (5 s) of the adrenal glands were obtained with the foals in a standing or sternal position, using a convex electronic transducer (4–5.5 MHz) (M-Turbo, FUJIFILM Sonosite, Bothell, WA, USA; MyLabAlpha, ESAOTE, Genova, Italy); the ultrasound technique described previously [40,41] was used. Videos were stored in DICOM format and retrospectively reviewed, in a blinded fashion, at the end of the season by a single observer (E.L.). Only frames allowing complete visualization of the medio-lateral and dorso-ventral diameter of the gland were chosen for each adrenal gland. The height of the whole adrenal gland and the width of the whole adrenal gland and medulla were measured as previously described [42]. The echogenicity of the cortex and medulla was subjectively assessed and recorded (Figure 1).

### 2.5. Hormone Concentrations Determination

Plasma ACTH, cortisol, DHEA, P4 and aldosterone concentrations were assessed. Plasma ACTH concentrations were determined using a commercial human-specific radioimmunoassay already used in horses (ACTH Radioimmunoassay, Beckman Coulter, Prague, Czech Republic) [42]. The reproducibility, measured as the intra- and inter-assay coefficients of variation, was 8.9% and 9.8%, respectively; the overall accuracy was 95%.

The concentrations of cortisol, DHEA and P4 were measured using a solid-phase microtiter RIA. The plasma samples were extracted with diethyl ether for cortisol and DHEA analysis, and petroleum ether for P4 analysis. In brief, a 96-well microtiter plate (Optiplate, Perkin-Elmer Life Science, Waltham, MA, USA) was coated with goat anti-rabbit γ-globulin serum diluted 1:1000 in a 0.15 mM sodium acetate buffer (pH 9) and incubated overnight at 4 °C. The plate was then washed twice with RIA buffer (0.05 M phosphate-buffered saline, pH 7.5, 0.1% BSA) and incubated overnight at 4 °C with 200 μL of the antibody serum diluted 1:20,000 for cortisol, 1:80,000 for DHEA and 1:8000 for P4. The cross-reactivity of the anti-cortisol antibody with other steroids was as follows: cortisol, 100%; prednisolone, 44.0%; prednisone, 4.3%; cortisone, 4.3%; corticosterone, 2.8%; 11-deoxycorticosterone, 0.7%; 17-hydroxyprogesterone, 0.6%; dexamethasone, 0.1%; progesterone, <0.01%; DHEA sulfate, <0.01%; pregnenolone, <0.01%. The cross-reactivity of the anti-DHEA antibody with other steroids was as follows: DHEA, 100%; 5-androsten-3β,17β-diol, 9.2%; epiandrosterone, 2.8%; pregnenolone, 1.8%; 5α-androstane-3β,17β-diol, 0.6%; cholesterol, 0.2%; testosterone, 0.1%; androstenedione, 0.1%; DHEA sulfate, 0.04%; cortisol, <0.001%. The cross-reactivity of the anti-progesterone antibody with other steroids was as follows: P4, 100%; 11 β-OH-progesterone, 46%; 17 α-OH-progesterone, 0.4%; 20 α-OH-progesterone, 0.04%; testosterone, 0.08%; cortisol, <0.01%; 17-β-estradiol, <0.01%; 17-α-estradiol, <0.01%; estrone, <0.01%. After washing the plate with RIA buffer, the standards (5–200 pg/well), the quality control extract, the test extracts and the tracer (hydrocortisone (cortisol [1,2,6,7-^3^H (N)]-), DHEA (1,2,6,7-^3^H N]), progesterone (1,2,6,7-^3^H [N])) were added and the plate was incubated overnight at 4 °C. The bound hormone was separated from the free hormone by decanting and washing the wells in RIA buffer. After the addition of 200 μL of a scintillation cocktail (Microscint 20, Perkin-Elmer Life Sciences, Waltham, MA, USA), the plate was counted on a β-counter (Top-Count; Perkin-Elmer Life Science, Waltham, MA, USA).

The intra- and inter-assay coefficients of variation were 3.7 and 10.1%, 3.8 and 10.6%, and 3.4 and 8.2%, for cortisol, DHEA and P4, respectively. The sensitivities of the assays were 1.35 pg/well, 0.62 pg/well and 0.56 pg/well for cortisol, DHEA and P4, respectively.

The relationships between plasma cortisol, plasma DHEA, plasma P4 and their respective standard curves, determined through linear regressions, were linear, with a correlation coefficient of *r* = 0.99. The models were described by the equations *y* = 0.923*x* + 3.398, *y* = 0.8869*x* + 1.312 and *y* = 0.9926*x* + 0.9884, for cortisol, DHEA and P4, respectively.

Plasma aldosterone concentration was determined using a commercial human-specific radioimmunoassay validated for equine samples (Active Aldosterone Radioimmunoassay, Beckman Coulter, Prague, Czech Republic) [43]. The reproducibility, measured as the intra- and inter-assay coefficients of variation, was 10.2% and 9.6%, respectively; the overall accuracy was 98%.

### 2.6. Statistical Analysis

All measurements were tabulated in a Microsoft Excel worksheet and transferred to R Studio (version 3.4.3) and JASP (version 0.8.6.0) statistical software for analysis.

The homoscedasticity of the variables was tested using the Shapiro–Wilk test (normal distribution) and Levene’s test (homogeneity of the variance), and subsequent tests chosen as appropriate.

Descriptive statistics, including the mean, SD or median (min/max values of the 25th and 75th percentiles) were used when appropriate. The median age and weight of the foals, the median age of the mares and the median pregnancy length were calculated for each breed. Differences between the Thoroughbred and Standardbred groups in age, weight, pregnancy length and age of the mares were assessed using the Mann–Whitney test. Fisher’s test was used to assess the presence of significant differences for sex between the Thoroughbreds and Standardbreds.

The mean value and the standard deviation were calculated for the measurements of the adrenal glands. For each measurement obtained, the correlation with the age and the weight of the foal was calculated using Pearson’s *r* coefficient. Student’s *t*-test was used to assess the presence of sex- and breed-related differences in the adrenal gland measurements.

The ACTH/cortisol, ACTH/DHEA, ACTH/P4, ACTH/aldosterone and cortisol/DHEA ratios were calculated. The Mann–Whitney test was used to assess the presence of breed-related differences in hormone concentrations. Hormone concentrations and ratios are expressed as the median and range (min–max).

Significance was set at *p* < 0.05.

## 3. Results

### 3.1. Foals

Ten healthy neonatal Thoroughbred (TB) and 10 healthy neonatal Standardbred (SB) foals were included in the study (8 colts and 12 fillies; age median: 31 h; age range: 7–72 h).

In the Thoroughbred group, the median age and weight of the foals on the day of ultrasound examination were 33 h (interquartile range (ir: 12–72 h) and 51.5 kg (ir: 46.5–61.2). Four out of 10 were fillies and six were colts; the pregnancy length and the median age of the mares were 342 days (ir: 337–347.7) and 14 years (ir: 9–15 years), respectively. Five out of 10 were born in March, three in April and two in May.

In the Standardbred group, the median age and weight of the foals at ultrasound examination were 30.5 h (ir: 7.7–58.7 h), 50 kg (ir: 45–55 kg). Eight out of 10 were fillies and two were colts; the length of pregnancy and age of the mares were 339.5 days (ir: 334.2–348.5) and 9.7 years (ir: 5.7–13.2 years), respectively. Two out of 10 foals were born in February, one in March, three in April and four in May.

All mares were multiparous. No significant difference was found in age (*p* = 0.85), weight (*p* = 0.385) and sex of the foals (*p* = 0.133), pregnancy length (*p* = 0.769) and mare’s age (*p* = 0.402) between the Thoroughbred and Standardbred groups.

### 3.2. Adrenal Gland Ultrasonographic Measurements

In both groups, the adrenal glands were well visualized at the 16th to 17th intercostal space, as previously described [40]. In all cases, the adrenal cortex and the medulla were judged to be normally hypoechogenic and echogenic, respectively. The measurement of the whole right and left adrenal gland height, the right dorsal and ventral medulla width, the whole right dorsal and ventral lobe width, and the whole left adrenal gland and medulla width [41] were obtained for both glands in all foals and are reported in Table 1.

No significant correlation was found between the ultrasonographic measurements and the age of the foals.

A moderate significant positive correlation was found only between the height of the right adrenal gland and the weight of the foals in the overall population (*p* = 0.03; *r* = 0.487).

No significant sex-related differences were found.

There were statistically significant breed-related differences: the width of the whole adrenal glands and the medullas bilaterally were significantly lower in Thoroughbred foals than in Standardbred ones (Table 1).

### 3.3. Hormone Concentrations and Hormone Ratios

Plasma hormone concentrations and hormone ratios are presented in Table 2. There was a significantly higher cortisol and lower ACTH concentration in Thoroughbred foals compared with Standardbred foals (*p* = 0.013). The cortisol/DHEA ratios in the Thoroughbred foals were significantly higher than in the Standardbred foals (*p* = 0.035). No other statistical differences in hormone concentrations were found between the two groups.

## 4. Discussion

In this study, ultrasonographic measurements of the adrenal glands were obtained and compared between a group of healthy neonatal Thoroughbred foals and a group of healthy neonatal Standardbred foals.

As previously described [40,44], all the evaluated adrenal glands were characterized by a hypoechogenic cortex and echogenic medulla in the healthy neonatal foals.

The normal ultrasonographic appearance, location and measurements of the adrenal glands have been described in healthy puppies and kittens. However, to the authors’ knowledge, no data about the effect of age, weight, sex and breed on ultrasonographic measurements of the adrenal glands have been reported in these individuals; consequently, a direct comparison between neonatal foals and neonatal puppies or kittens cannot be made [45,46].

In contrast with reports in healthy adult dogs [35], no significant correlation was found between adrenal gland measurements and the age of the foals. This different result is likely due to the inclusion of only neonates in the present study, implying that a narrower range of age was considered. However, in the authors ‘opinion, the absence of a correlation with age detected in this study and the lack of variation in the ultrasonographic measurements of the adrenal glands reported in the first 5 days of life may encourage the use of the same reference ultrasonographic measurement range for healthy foals of any age within 5 days of life in clinical evaluations [41]. Further studies including a wider range of age are needed. A significant moderate positive correlation between body weight and the measurements was found only for the height of the right adrenal gland. In dogs, Mogicato and colleagues found a significant effect of body weight only for the length of both adrenal glands (the greater the body weight, the greater the length). The measurements not affected by body weight were considered to be more useful in clinical evaluations, and the same could be true for neonatal foals [36]. Later, Soulsby and colleagues found a weak to moderate correlation between body weight and all the measurements considered; they concluded that ultrasonographic adrenal gland measurements can vary significantly based on body weight and they proposed the use of different normal cut-off values for different weight categories [38]. The absence of a correlation between body weight and the majority of ultrasonographic measurements in this study could be related to the different ultrasonographic planes used to perform adrenal gland measurements in foals compared with dogs [36,38]; furthermore, the low number of foals and the narrow range of weight considered could have influenced the absence of a correlation and a different result can be expected in a different population with a wider range of weights.

No significant sex-related differences in the measurements of the adrenal glands were found in this study, in accordance with other previous reports in dogs and cats [35,45].

Mogicato et al. [36] found a weak effect of sex on the ultrasonographic size of the adrenal gland in dogs but they considered the result to be a random effect because of the impossibility of finding a physiologic explanation.

Interestingly, the widths of the whole glands and medulla on both sides were all significant higher in the Standardbreds. To the authors’ knowledge, no breed-related differences in adrenal gland measurements in other species have been reported in the literature, if these are not secondary to weight differences. It can be speculated that other breed-related factors may have influenced the results. Significant differences in abdominal circumference, total body weight and height were detected between Friesian and Warmblood adult horses, and have been proposed to potentially influence the diameter and the area of multiple arterial vessels in a recent study by Vera and colleagues [46]. It could be speculated that the same may happen for measurements of the adrenal glands; however, unfortunately, these parameters were not measured in the present study and although a different conformation and size between Thoroughbred and Standardbred foals can be subjectively estimated, no objectives values can be provided by the authors. Importantly, the absence of a comparison between the ultrasonographic and the gross size of the adrenal glands is a limitation of the present study. Considering the small size of the adrenal glands and their deep localization in the abdomen, a significant discrepancy between ultrasonographic and anatomical size may be possible; it cannot be excluded that a different conformation or the size of the foal could induce a subtle difference in the ultrasonographic window used in the two breeds, which would be responsible for different ultrasonographic measurements of the adrenal glands.

Other variables such as the age and sex of the foal, pregnancy length and the mare’s age did not significantly differ between the two groups, and they are not likely to have influenced the results. Further studies are needed to assess whether ultrasonographic measurements of the adrenal glands correspond to gross anatomical differences between neonatal Thoroughbred and Standardbred foals, and any factor potentially responsible for the difference detected in this study.

Along with ultrasonographic measurements of the adrenal glands, hormone concentrations were assessed with the double purpose of assessing HPA axis function and comparing healthy neonatal Thoroughbred with Standardbred foals.

The cortisol and P4 plasma concentrations and hormone ratios obtained in this study in both groups were within the ranges obtained in healthy neonatal foals in previous studies; DHEA, ACTH and aldosterone concentrations slightly differed from those reported in the literature [2,15,20,47]. However, the values recorded for DHEA, ACTH and aldosterone did not reach the ranges reported in the literature even for sick neonatal foals [12,15,20,47]. Specifically, the median values obtained for DHEA were higher than the median value reported in the literature (7.7 ng/mL (not detectable: 117.9)), but still not as high as those reported for sick (101.1 ng/mL (not detectable: 1412.6)) and maladjusted neonatal foals (92.9 ng/mL (not detectable: 1511)) [47]. The aldosterone and ACTH values were lower than those reported previously in healthy foals (185.3 pg/mL (20–1175.7); 24.4 pg/mL (10–279)), and were even lower than those reported in sick non-septic (204.5 pg/mL (16.8–1548); 45.7 pg/mL (10–1250)) and septic (357.6 pg/mL (18.7–2233)) foals [20]. In the authors’ opinion, these differences could be due to the different methods and/or producers of the commercial test kits used: in this study, ACTH and DHEA were assessed using RIA instead of immunochemiluminescence and liquid chromatography mass spectrometry [20,47], while aldosterone concentration was determined by using a commercial RIA kit that was different from that used by Dembek et al. [20].

In light of this, and considering the low ACTH/steroids ratios characterizing the healthy neonatal foals of this study, normal functionality of the adrenal glands in these foals can be inferred [2,15].

Interestingly, in Thoroughbred foals the cortisol and ACTH concentrations were significantly higher and lower, respectively, in comparison to Standardbreds.

In healthy foals, Castagnetti at al. [15] found a significantly higher ACTH concentration at birth than at 12 and 24 h of life and a significantly higher cortisol concentration at birth than at 24 h. Similarly, the P4 concentrations in healthy neonatal foals have been reported to be high at birth and to decrease by the end of the first day of life, falling to near zero at 2 days postpartum in healthy foals [48,49,50].

Despite an age-related variability of some of the steroid hormones has been described, no statistically difference was found between the age of the two groups and it is unlikely that age could have affected the difference in ACTH and cortisol concentration between the two groups.

The majority of Thoroughbred foals were born between March and April; in contrast, the majority of Standardbred foals were born in April and May. It can be speculated that the different temperature and/or light conditions may have influenced the hormone concentrations. In a previous study, where the effect of environmental factors such as temperature, rainfall and light conditions on hair cortisol concentration in neonatal foals was investigated, none of the factors evaluated was demonstrated to influence the cortisol concentration [51]; however, since plasma and hairs represent two different matrices, they provide different information on cortisol concentration and, consequently, the influence of environmental factors on cortisol plasma concentration in healthy foals cannot be completely excluded in this study. We can also hypothesize that, as they were born at the Perinatology Unit of the University of Bologna, the Standardbred foals were more accustomed to close contact with humans and that the clinical examination and blood sampling represented a lesser source of stress than in the Thoroughbred foals. In contrast, in a study reported by Hart et al. [52], no differences in cortisol concentrations were found between healthy stressed and healthy non-stressed neonatal foals. Other variables such as the sex of the foal, the length of pregnancy and the age of the mare did not significantly differ between the two groups, and they are not likely to have influenced the results. Although the influence of other confounding factors associated with management or environment cannot be completely excluded, the higher cortisol concentrations in Thoroughbred foals of our study may be explained by the presence of a higher sensitivity to stress in this breed instead of a higher source of stress.

In support of this, in a previous study in which the effect of the weaning was investigated in Thoroughbred, Standardbred and German Warmblood foals, no significant differences were found in cortisol concentrations but, on the basis of the hematological and metabolic parameters, Thoroughbred foals were considered toe the most sensitive to weaning-induced stress; however, the study was focused only on foals 5–6 months of age instead of neonatal foals [53].

Cortisol/DHEA ratios were also investigated in the present study. Both cortisol and DHEA are considered indicators of allostatic load [54,55] and resilience [22,56], and their ratio is believed to be an index of anabolic/catabolic balance [57]. The ratio can be an important factor in determining how an individual’s HPA axis is functioning [58,59,60]. In adult pigs, Trevisan et al. [23] observed that an increased cortisol/DHEA ratio suggested that an elevated metabolic effort was needed to cope with the environment in terms of housing, feeding and re-grouping. In milking cows at pasture, a deterioration in environmental conditions corresponded to an increase in the cortisol/DHEA ratio in the last part of summer [22]. In light of that, in our study, the different cortisol/DHEA ratios in the two groups may suggest a greater resilience (and ability to cope with the stressors) in Standardbred compared with Thoroughbred foals.

In this study, the correlation between adrenal gland ultrasonographic measurements and hormone concentrations was not tested statistically. Interestingly enough, when the percentage of the whole gland width occupied by the medulla in ultrasonographic images was compared in the two groups, a trend for the values of Standardbred foals to be higher than those of Thoroughbred foals (68% vs. 61%, 69% vs. 64% and 68 vs. 66% for the right dorsal lobe, right ventral lobe and left gland, respectively) was found, suggesting that if a larger proportion of the gland is made of medulla, less cortex is available. Although this represents only a trend, it can be speculated to be related to glucocorticoid production and, consequently, to differences in the hormone concentrations detected in the two groups, and this should be more thoroughly investigated in future. Any correlation between the ultrasonographic appearance and measurements of the adrenal glands and hormone concentrations warrants further insight.

Two main limitations can be recognized in this study: first of all, two different operators obtained the images in the two groups although the repeatability of the image acquisition has not been tested; moreover, two different ultrasonographic machines were used for the study. Considering the first limitation, the operator-dependent variability in image collection could have potentially affected the results. However, all the operators had the same training and, furthermore, one single observer selected the best images from the videos and performed the measurements. In light of this, in the authors’ opinion, the variability linked to image collection and its effects on the final results are limited.

The use of different ultrasound machines could not be avoided, since the study involved two different veterinary hospitals. Despite the two guaranteeing high-quality images, differences in the images’ resolutions cannot be completely excluded. The images’ settings and quality were set to be as similar as possible between the two ultrasonographic devices: the depth setting was fixed at 11 cm and maintained for all the foals, the focus position was adjusted at the level of the adrenal gland and gains were adjusted to allow optimization of the image, defined as the ability to correctly identify the adrenal gland from the surrounding tissue.

## 5. Conclusions

This study provides ultrasonographic measurements of the adrenal glands and adrenal/HPA axis hormone concentrations for healthy neonatal Thoroughbred and Standardbred foals. The differences found in ultrasonographic measurements between neonatal Standardbred and Thoroughbred foals likely represent breed-related differences, suggesting that breed-specific reference ranges might be needed in view of clinical applications.

Beside these findings, the differences in cortisol and ACTH concentrations and the cortisol/DHEA ratio detected in the two groups potentially reflect a different allostatic load and resilience between the two breeds, which might suggest different responses to stress and possibly illness. This warrants investigation in a different setting of the HPA axis, where, in particular, environmental and management influences can be excluded.

Further studies with larger populations are needed to confirm breed-related differences in adrenal ultrasonographic sizes and hormone concentrations, and to investigate any correlation between hormone concentrations and ultrasonographic measurements of the adrenal glands.

## Figures and Tables

**Table 1:** Objective ultrasonographic measurements of adrenal glands, aorta and caudal cava vein in healthy neonatal Standardbred (*n* = 10) and Thoroughbred (*n* = 10) foals (≤72 h). Results are expressed as the mean value and standard deviation.

Measurement (cm)	Standardbred	Thoroughbred	*p*-Value
Whole right adrenal gland height	3.21 ± 0.41	3.02 ± 0.86	0.577
Whole left adrenal gland height	2.92 ± 0.60	2.7 ± 0.79	0.517
Right dorsal medulla width	0.56 ± 0.09	0.38 ± 0.15	0.007 *
Whole right dorsal lobe width	0.82 ± 0.09	0.60 ± 0.16	0.003 *
Right ventral medulla width	0.57 ± 0.09	0.36 ± 0.15	0.002 *
Whole right ventral lobe width	0.84 ± 0.11	0.56 ± 0.19	0.002 *
Left medulla width	0.61 ± 0.13	0.42 ± 0.2	0.02 *
Whole left adrenal gland width	0.83 ± 0.10	0.61 ± 0.18	0.005 *

* Significant difference in the row.

**Table 2:** Plasma hormone concentration and ratios expressed as the median and range in healthy neonatal Standardbred (*n* = 10) and Thoroughbred (*n* = 10) foals (≤72 h).

Variables	Standardbred (Mean Age, 30.5 h)	Thoroughbred (Mean Age, 33 h)	*p*-Value
ACTH (pg/mL)	8.9 (6.17–12.6)	4.75 (3.67–8)	0.023 *
Cortisol (ng/mL)	11.50 (7.8–15.68)	21.38 (18.7–31.37)	0.013 *
DHEA (ng/mL)	38.82 (31.26–101.90)	18.6 (12.9– 30.43)	0.353
P4 (ng/mL)	3.07 (1.81–6.33)	4.6 (3.1–5.82)	0.393
Aldosterone (pg/mL)	21.4 (14.7–60)	19.25 (8.7–47.7)	0.684
ACTH/Cortisol Ratio	0.58 (0.22–0.94)	0.24 (0.14–0.37)	0.070
ACTH/P4 Ratio	4.4 (0.92–5.82)	1.2 (0.95–2.04)	0.063
ACTH/Aldosterone Ratio	0.44 (0.09–1)	0.29 (0.9–2.04)	0.623
ACTH/DHEA Ratio	0.34 (0.05–0.98)	0.23 (0.21–0.47)	0.705
Cortisol/DHEA Ratio	0.32 (0.14–0.62)	1.27 (0.86–1.56)	0.035 *

DHEA, dehydroepiandrosterone; ACTH, adrenocorticotropin; P4, progesterone. * Significant difference in the row.

## Data Availability

The data presented in this study are available in the Supplementary Materials.

## Supplementary Materials

The following are available online at https://www.mdpi.com/article/10.3390/ani11061832/s1, Table S1: Dataset.
